# Correction: Nanobodies: site-specific labeling for super-resolution imaging, rapid epitope-mapping and native protein complex isolation

**DOI:** 10.7554/eLife.15597

**Published:** 2016-03-16

**Authors:** Tino Pleiner, Mark Bates, Sergei Trakhanov, Chung-Tien Lee, Jan Erik Schliep, Hema Chug, Marc Böhning, Holger Stark, Henning Urlaub, Dirk Görlich

Pleiner T, Bates M, Trakhanov S, Lee CT, Schliep JE, Chug H, Böhning M, Stark H, Urlaub H, Görlich D. 2015. Nanobodies: site-specific labeling for super-resolution imaging, rapid epitope-mapping and native protein complex isolation. *eLife* **4**:e11349. doi: 10.7554/eLife.11349.

Published December 3, 2015

An error was identified in Figure 6d (bottom right panel). The image of XL177 cells stained with the Alexa Fluor 647 maleimide-labeled anti-Nup93 nanobody mistakenly duplicated the neighbouring Nup98 image. The error occurred during figure assembly in Adobe Illustrator.

The corrected Figure 6 is shown here:

**Figure 1:**
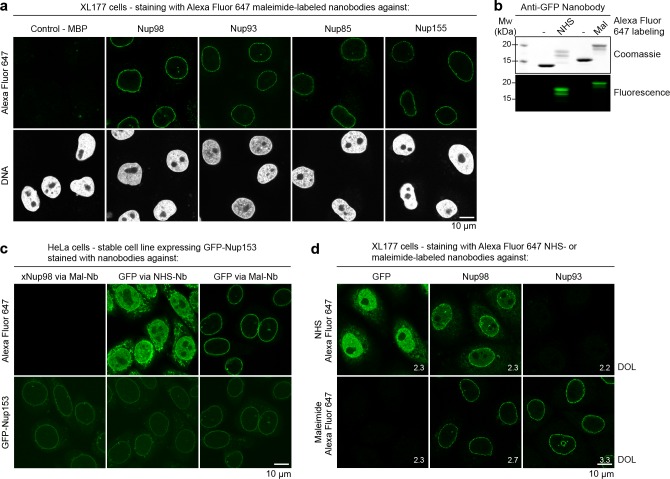

The originally published Figure 6 is also shown for reference:

**Figure 2:**
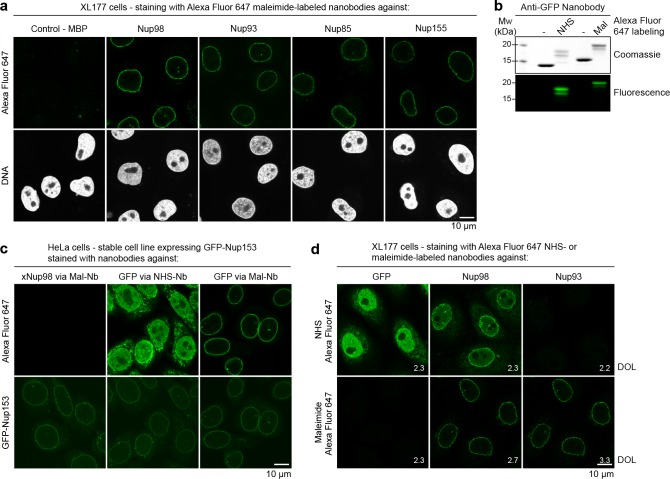

The article has been corrected accordingly.

